# PANoptosis as a Two-Edged Sword in Colorectal Cancer: A Pathogenic Mechanism and Therapeutic Opportunity

**DOI:** 10.3390/cells14100730

**Published:** 2025-05-16

**Authors:** Györgyi Műzes, Ferenc Sipos

**Affiliations:** Immunology Division, Department of Internal Medicine and Hematology, Semmelweis University, 1088 Budapest, Hungary

**Keywords:** PANoptosis, colorectal cancer, PANoptosome, apoptosis, pyroptosis, necroptosis, tumor microenvironment, ZBP1, RIPK3, CASP8

## Abstract

The examination of PANoptosis in colorectal cancer is particularly important, as many tumor cells can evade apoptotic cell death while continuing to proliferate through inflammatory mediators and creating an immunosuppressive environment. The PANoptosome functions as a regulatory complex that unites proteins governing pyroptotic, apoptotic, and necroptotic pathways, rather than allowing distinct death pathways to compete. The expression and functional status of key molecules within the PANoptosome, such as ZBP1, RIPK1, RIPK3, CASP8, and ASC, may influence tumor viability and immune detection. The tumorigenic impact of PANoptosis is complex and predominantly manifests through chronic inflammation, immune response modulation, and changes in the tumor microenvironment. PANoptosis also aids in the defense against colon cancer by directly eradicating tumor cells and modifying the cellular environment. The expression profile of PANoptosis components may possess prognostic and predictive significance. The therapeutic ramifications of PANoptosis in colorectal cancer are now being investigated through many avenues. It provides an opportunity to develop targeted therapeutic techniques. In contrast, it may also be pertinent in conjunction with immunotherapy, as PANoptosis signifies an immunogenic type of cell death and may consequently enhance the anti-tumor immune response. A thorough comprehension of how these parameters influence PANoptosis is crucial for practical implementation.

## 1. Introduction

Colorectal carcinoma (CRC) ranks among the foremost causes of cancer mortality globally, with inflammatory mechanisms, the tumor microenvironment, and the dysregulation of apoptosis significantly contributing to its growth and progression [1]. Multiple treatment options exist for CRC, encompassing chemotherapy and immunotherapy [2]. Regrettably, clinical outcomes continue to be suboptimal due to tumor heterogeneity, hereditary characteristics, and many risk factors [3,4]. About 25% of CRC patients are discovered at an advanced stage, and nearly 50% of those diagnosed at an early stage subsequently develop metastatic disease [5,6]. The 5-year survival rate for individuals with minimal metastatic lesions is 40% after surgical resection and systemic therapy, while for those with advanced metastatic CRC, the survival rate significantly decreases (approx. 20%) [7,8]. Given the low survival rate of CRC patients, there is a need to establish a precise classification of the disease that might enhance the prediction of patient outcomes and responses to immunotherapy and chemotherapy, thereby forming tailored treatment regimens and increasing prognoses.

Programmed cell death (PCD) is conventionally categorized into five primary forms: apoptosis, autophagy, necroptosis, pyroptosis, and ferroptosis [9]. A recent study has revealed a novel, integrated form of cell death that amalgamates the pathways of pyroptosis, apoptosis, and necroptosis, termed PANoptosis [10]. The PANoptosome, a multifunctional protein complex, facilitates PANoptosis by simultaneously activating caspases, gasdermin D, and necroptotic kinases [11].

The functions of apoptosis, pyroptosis, and necroptosis in oncology research are areas of vigorous discussion. The investigation of PANoptosis in CRC is notably significant since numerous tumor cells can circumvent apoptotic cell death while sustaining proliferation via inflammatory mediators and fostering an immunosuppressive milieu [12]. The expression and functional status of critical molecules comprising the PANoptosome, including ZBP1, RIPK1, RIPK3, CASP8, and ASC, can have a bidirectional impact on tumor survival, immune recognition, or tumor cell death [13]. The existence or nonexistence of these compounds may hold prognostic significance for treatment, particularly in immunotherapeutic strategies.

Considering the dual function of PANoptosis in either inhibiting or facilitating colorectal cancer, comprehensive knowledge of its mechanisms could reveal essential elements of tumor biology and pave the way for innovative diagnostic and therapeutic approaches in an era increasingly characterized by precision oncology. Targeted manipulation of PANoptosis, such as triggering immunogenic cell death in tumor cells, may provide novel therapeutic avenues for colorectal cancer treatment.

This review aims to elucidate the pathogenic and therapeutic roles of PANoptosis in CRC, with an emphasis on preclinical and clinical relevance.

## 2. Fundamental Characteristics and Processes of PANoptosis

PANoptosis is a multifaceted, inflammatory PCD that encompasses aspects of apoptosis, necroptosis, and pyroptosis; however, it cannot be solely classified as any one of these forms [14,15]. The mechanism is governed by a multi-protein complex known as the PANoptosome, which comprises essential components from all three cell death pathways.

It is crucial to differentiate PANoptosis from other forms of cell death. Apoptosis is a non-inflammatory, so-called immunologically silent form of cell death characterized by the activation of caspases (e.g., CASP3, -8, and -9) and is associated with phenomena such as cell disintegration (blebbing) [16]. RIPK1-RIPK3-MLKL is involved in necroptosis, an inflammatory form of cell death that leads to the cell membrane becoming permeable [17]. Pyroptosis is a form of inflammatory cell death characterized by the activation of inflammatory caspases (e.g., CASP1, -4, -5, and -11) and results in the creation of gasdermin pores [14]. The PANoptosome orchestrates PANoptosis, which integrates the mechanisms of apoptosis, necroptosis, and pyroptosis, typically in response to a singular stimulus. This type of cellular demise is particularly significant in infections, autoimmune disorders, and tumors, as it initiates a complicated immunological response [18]. The activation of PANoptosis involves a coordinated action of multiple molecular players, as detailed below.

The PANoptosome is a supramolecular signaling complex that unites essential components from pyroptotic, apoptotic, and necroptotic pathways [19]. Pathogen-associated molecular patterns (PAMPs), damage-associated molecular patterns (DAMPs), or cytokine signaling (e.g., tumor necrosis factor/TNF/, IFNs, and IL-1) initiate the assembly [20].

The assembly of the PANoptosome is mostly reliant on scaffold proteins, including Z-DNA binding protein 1 (ZBP1), apoptosis-associated speck-like protein containing a CARD (ASC), and receptor-interacting serine/threonine-protein kinase 3 (RIPK3) [21,22]. Upon assembly, the PANoptosome binds and activates numerous cell death effectors, such as caspases (CASP1, -3, and -8), gasdermins (GSDMD, GSDME), and mixed lineage kinase domain-like pseudokinase (MLKL), leading to a coordinated execution of cell death pathways [23].

**Figure 1:**
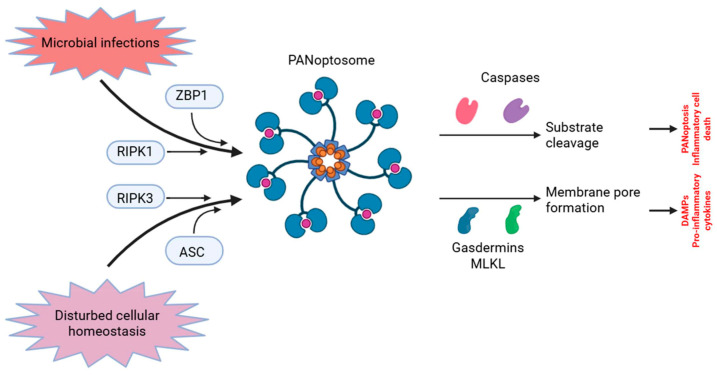
The process of PANoptosome assembly and the initiation of PANoptosis. In response to cellular damage, sensors can identify the disruption and trigger the development of the PANoptosome. PANoptosomes possess the capacity to integrate many elements from previously distinct cell death processes. The activation of caspases, gasdermins, MLKL, and others results in substrate cleavage and the development of cell membrane pores, facilitating the release of DAMPs and pro-inflammatory cytokines. ASC: apoptosis-associated speck-like protein containing a caspase activation and recruitment domain; DAMP: danger-associated molecular pattern; RIPK: receptor-interacting serine/threonine protein kinase; MLKL: mixed lineage kinase domain-like pseudokinase; ZBP1: Z-DNA-binding protein 1. Figure was partly created with https://biorender.com/ (accessed on 5 April 2025).

Caspases, RIPK3, gasdermins, and ZBP1 are essential activating molecules [24]. When activated by the inflammasome, caspase-1 cleaves the pro-inflammatory cytokines IL-1β and IL-18, and GSDMD, which leads to the formation of pyroptotic pores [24]. CASP3 and CASP7 are quintessential executors of apoptosis, cleaving substrates such as poly(ADP-ribose) polymerase (PARP) [25]. CASP8 operates at the intersection of apoptosis and necroptosis. It can either cause apoptosis through death receptors or inhibit necroptosis by blocking RIPK3/MLKL activation [25]. RIPK3 is a crucial regulator of necroptosis that interacts with RIPK1 and MLKL [26]. In the absence or inhibition of caspase-8, RIPK3 phosphorylates MLKL, resulting in plasma membrane rupture and necroptotic cell death [27]. In PANoptosis, RIPK3 interacts with ZBP1, promoting the assembly of the PANoptosome [28]. GSDMD is cleaved by caspase-1 or caspase-11, resulting in the formation of membrane holes indicative of pyroptosis. GSDME is cleaved by CASP3, transforming apoptosis into secondary pyroptosis and enhancing inflammatory responses [29]. ZBP1 functions as a crucial PANoptotic sensor in reaction to viral infections [30]. It associates with Z-form nucleic acids, attracting RIPK3 and initiating PANoptosome formation. It can induce all three types of cell death by activating RIPK3, CASP8, and CASP1 [30] (Figure 1).

The equilibrium among these molecules dictates the prevailing mechanism of cellular demise. Under typical circumstances, caspase-8 inhibits necroptosis and pyroptosis, thereby averting severe inflammation [31]. During infections or cytokine storms, PANoptosis functions as a fail-safe mechanism to eradicate diseased or malignant cells [32]. Table 1 delineates the principal molecular constituents implicated in PANoptosis pathways.

## 3. Synergistic and Evasive Functional Effects of the PANoptosome as a Central Integrative Hub

**Figure 2:**
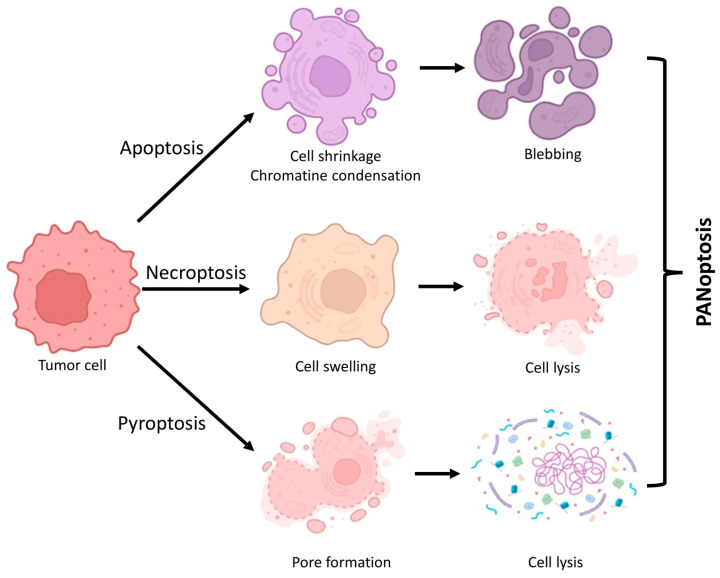
Cellular attributes of PANoptosis. PANoptosis is a type of inflammatory programmed cell death that incorporates molecular components of apoptosis, necroptosis, and pyroptosis. The schematic image illustrates the cellular changes associated with the primary kinds of cell death. Figure was partly created with https://biorender.com/ (accessed on 5 April 2025).

It is evident that PANoptosis incorporates elements from several cell death mechanisms, with synergies and overlaps facilitating multiple routes to attain the ultimate biological response. Instead of separate death pathways competing with each other, the PANoptosome works as a regulatory complex that brings together proteins that control pyroptotic, apoptotic, and necroptotic processes (Figure 2). Scaffold proteins, including ZBP1, RIPK3, and ASC, facilitate the recruitment and activation of necessary effectors. Depending on the cellular setting (e.g., infection, cytokine storm, or malignancy), many molecules are activated to trigger overlapping pathways of cell death [33]. In viral infections, the detection of cytosolic viral nucleic acids by sensors such as ZBP1 or AIM2 can result in the robust assembly of PANoptosomes and the demise of inflammatory cells [34]. In contrast, the expression or activation state of PANoptosis regulators (e.g., ZBP1, RIPK3, and caspase-8) can be altered by factors such as hypoxia, metabolic stress, and DNA damage in the tumor microenvironment, potentially suppressing or reprogramming PANoptosome formation [35]. Consequently, the availability of upstream signals, post-translational modifications, and interaction partners that regulate PANoptosome activation and outcome is determined by the cellular context. If one pathway is obstructed, PANoptosis guarantees cell death by transitioning to other mechanisms [36].

The majority of the redundancy provided by PANoptosis is intended for fail-safe functionality. Apoptosis is inhibited in numerous malignancies and viral infections [37,38,39,40,41,42]. In these instances, pyroptosis and necroptosis serve as compensatory mechanisms for the removal of compromised cells [43,44,45].

Another important goal or consequence of functional synergism is to increase inflammation. In contrast to apoptosis, which is typically immunologically silent [46], PANoptosis provides a robust immune response through the activation of DAMPs, cytokines, and the inflammasome [32].

PANoptosis enhances host defense and facilitates effective pathogen eradication. Numerous infections strive to obstruct a specific type of cell death; however, PANoptosis mitigates the effect by concurrently activating several pathways. In mammals, innate immunity serves as the primary defense against viral infections. After getting infected, host pattern recognition receptor (PRR) systems sense viral PAMPs. Toll-like receptors (TLRs), C-type lectin receptors (CLRs), NOD-like receptors (NLRs), RIG-I-like receptors (RLRs), and AIM2-like receptors (ALRs) comprise these systems. The identification of viral PAMPs by PRRs initiates the activation of innate immune signaling pathways, including the transcription factor NF-κB and MAPK signaling, leading to the production of inflammatory cytokines and interferons, thereby preparing the immune response [47]. The activation of PRRs frequently results in diverse types of cellular death. The eradication of infected host cells through PCD death pathways is essential for halting viral dissemination. Conversely, at the organismal level, cellular apoptosis can exacerbate disease pathogenesis during viral infections; inflammatory byproducts (e.g., DAMPs, alarmins, supplementary PAMPs, and inflammatory cytokines) are released from necrotic cells, leading to a cytokine storm, organ damage, and death [48,49,50]. Consequently, the time and degree of cell death activation are meticulously and intricately regulated under optimal conditions, largely to safeguard the host [51].

The primary objective is to eradicate the infected cells to ensure the host’s survival. Intracellular pathogens, including viruses (e.g.,: influenza A virus, murine hepatitis virus, vesicular stomatitis virus, herpes simplex virus 1, −2, or human cytomegalovirus), can circumvent this by obstructing or redirecting the pathways for host cell death, facilitating their own proliferation [52,53,54,55,56,57]. The virus-induced suppression of a specific PCD pathway may evolutionarily foster the development of mechanisms that enhance alternative cell death executors and effectors via a shared signaling framework. This highlights the significance of comprehending the interconnections among PCD pathways.

Not only viruses, but also bacteria, have evolved mechanisms to bypass the immune response to PANoptosis, thus facilitating their survival and spread [58]. Shigella concurrently manipulates the interplay of apoptosis, necroptosis, and pyroptosis to undermine the host’s integrated defenses. Shigella disrupts these pathways by targeting CASP8 and RIPK1/3. Bacterial effector proteins, such as OspC1 and OspD3, inhibit apoptosis and necroptosis, respectively. OspC1 inhibits CASP8, preventing apoptosis while simultaneously facilitating RIPK1/RIPK3 signaling, which promotes necroptosis via the activation of MLKL. Conversely, OspD3 proficiently inhibits necroptosis by facilitating the degradation of RIPK1 and RIPK3, thereby disrupting their interaction with apoptosis [59]. Moreover, Shigella obstructs CASP4 activation via OspC3, thereby preventing pyroptosis [60]. Additional effectors, including the virulence factor VirA and the invasion plasmid gene D, further inhibit apoptosis via distinct pathways [61,62,63].

These processes underscore the intricate tactics utilized by pathogens to exploit host cell signaling and ensure survival. By evading scheduled cell death pathways, pathogens not only improve their own survival but also facilitates the endurance of the infection, presenting a considerable challenge to host immune responses. PANoptosis is not merely the presence of apoptosis, necroptosis, and pyroptosis; it is a highly integrated and synergistic process in which molecular effectors from distinct pathways cross-activate and enhance each other.

PANoptosis also contributes to the host’s antitumor immune defense by inducing a highly inflammatory form of cell death [13]. This process promotes the release of DAMPs and pro-inflammatory cytokines, enhancing dendritic cell activation, antigen presentation, and cytotoxic T cell priming [13]. Unlike apoptosis alone, PANoptosis can bypass tumor resistance by simultaneously activating multiple death pathways, leading to effective tumor cell elimination. The resulting inflammatory microenvironment recruits innate and adaptive immune cells, further amplifying immune responses [13]. Moreover, components of the PANoptosome, such as ZBP1 and AIM2, act as innate immune sensors that detect tumor-derived stress signals, initiating cell death and inflammation [21]. PANoptosis may also synergize with immunotherapies by converting immunologically “cold” tumors into “hot,” inflamed ones, thereby improving therapeutic responsiveness [64].

This integrated process guarantees strong immune activation, efficient pathogen or tumor cell elimination, and a reliable cell death response in inflammatory and disease contexts.

## 4. PANoptosis in Colorectal Tumor Development

### 4.1. Protumor Effects

**Figure 3:**
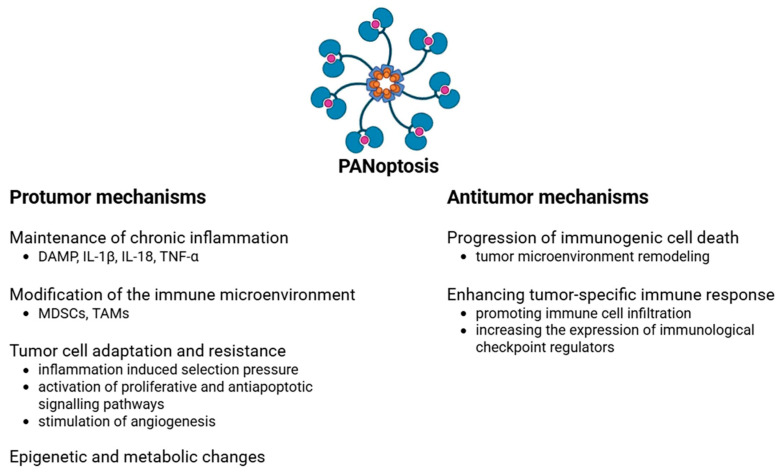
The protumor and antitumor effects of PANoptosis in colorectal cancer. DAMP: danger-associated molecular pattern; IL: interleukin; TNF: tumor necrosis factor; MDSC: myeloid-derived suppressor cell; TAM: tumor-associated macrophage. Figure was partly created with https://biorender.com/ (accessed on 5 April 2025).

The tumorigenic effect of PANoptosis is intricate and primarily occurs through chronic inflammation, immune response regulation, and alterations in the cellular environment (Figure 3). DAMPs and pro-inflammatory cytokines (e.g., IL-1β, IL-18, and TNF-α) generated during PANoptosis can provoke persistent inflammation, thereby facilitating tumorigenesis [65]. IL-1β and IL-18 can activate the NF-κB and STAT3 signaling pathways, thereby promoting cell proliferation and the production of anti-apoptotic proteins in colonic tumors [65]. The elevated production of reactive oxygen species (ROS) during chronic inflammation may result in mutations in the DNA of colonic epithelial cells [66,67,68]. These findings may partially elucidate the heightened risk of CRC in chronic inflammatory colitis.

PANoptosis can also modify the tumor immune microenvironment. Myeloid-derived suppressor cells (MDSCs) are pivotal in the advancement of colitis-associated CRC [69]. MDSCs facilitate tumor growth by augmenting angiogenesis, fostering chronic inflammation, and establishing a tumor microenvironment (TME) that inhibits immune system function [70]. Tumor-associated macrophages (TAMs) fulfill their critical functions by enhancing tumor proliferation, invasion, and migration; promoting angiogenesis; inhibiting antitumor immunity; altering metabolic profiles; and engaging with colonic microbiota [71]. The molecular components of PANoptosis can stimulate the immunomodulatory activities of MDSCs and TAMs, thereby diminishing the anti-tumor immune response [72,73,74,75,76,77,78].

Long-lasting chronic inflammation can lead to T cell exhaustion in colorectal cancer [79,80]. In this case, tumor antigens and inhibitory receptors like PD-1, PD-L1, CTLA-4, LAG-3, and Tim-3 are present [79,80]. This is especially true in MSI-H colorectal cancers with a high immunoscore [81]. T-cell exhaustion can result from the cytokines released during inflammatory cell death, which in turn reduces the elimination of tumor cells [82,83]. In the chronic inflammatory environment generated by PANoptosis, inflammation can even form an immunosuppressive tumor microenvironment [78,84].

PANoptosis-generated inflammatory cytokines in the TME may impose selection pressure on tumor cells, facilitating the survival of resistant clones and advancing disease progression. Elevated levels of IL-6 have been linked to resistance against chemotherapeutic treatments in CRC. IL-6 may stimulate the STAT3 signaling pathway, hence enhancing tumor cell survival and proliferation, which contributes to the emergence of resistant clones [85]. The persistent presence of TNF-α in the TME may enhance tumor cell resistance to apoptosis. This cytokine can stimulate the NF-κB pathway, thereby enhancing the production of anti-apoptotic genes, which in turn leads to the selective survival of resistant cells [86,87]. Prolonged exposure to IL-1β may enhance the invasiveness of colon cancer cells and facilitate treatment resistance. IL-1β-induced inflammatory responses may facilitate the adaptability of tumor cells to adverse environments, promoting the emergence of resistant clones [88].

While PANoptosis seeks to eradicate infected or damaged cells, it is conceivable that certain cancer cells may persist, triggering alternative signaling pathways that enhance proliferation and anti-apoptotic processes. Inflammatory cytokines generated during PANoptosis, including IL-1β and IL-18, can stimulate the NF-κB and STAT3 signaling pathways in adjacent cells. These pathways facilitate cellular survival and proliferation by augmenting the expression of anti-apoptotic proteins, including Bcl-2 and Bcl-xL [89]. Growth factors generated in an inflammatory milieu, such as VEGF and TGF-β, may also facilitate the proliferation of surviving cells and inhibit apoptosis [90,91]. These substances may activate the ERK/MAPK and PI3K/Akt signaling pathways, which are crucial for the regulation of cell growth and survival [90,91]. The consequence of all this is that tumor cells that survive in an inflammatory environment may become more resistant to immune responses and therapies.

Inflammatory cell death, especially pyroptosis, can substantially influence the metabolism of adjacent cells, notably enhancing glycolysis, which may facilitate tumor proliferation in colorectal cancer [92]. Inflammatory cytokines generated during pyroptosis, including IL-1β and IL-6, can activate the STAT3 and NF-κB signaling pathways in adjacent cells [93]. These pathways can enhance the production of glycolytic enzymes, thereby augmenting glucose absorption and metabolism in tumor cells [94]. Enhanced glycolysis facilitates the Warburg effect, whereby glucose is converted to lactate even in the presence of oxygen and pyruvate does not enter the oxidative phosphorylation pathway. Thus, despite the availability of oxygen, tumor cells produce energy predominantly via glycolysis. This metabolic alteration also facilitates accelerated cell division and tumor proliferation in colorectal cancer [95].

Epigenetic modifications, such as DNA methylation and histone alterations, that transpire during inflammation may exert enduring impacts on the behavior of tumor cells, particularly those of colon cancer. In chronic inflammatory situations, epigenetic modifications may enhance oncogene expression [96,97,98]. Consequently, focused manipulation of PANoptosis, such as anti-inflammatory methods and inflammasome inhibitors, may provide a viable therapeutic approach in anticancer treatment.

### 4.2. Antitumor Effects

PANoptosis contributes to the defense against colon cancer by directly eliminating tumor cells and altering the tumor microenvironment.

PANoptosis contributes to the progression of immunogenic cell death. In PANoptosis, tumor cells experience lysis, resulting in the release of cellular proteins, such as tumor cell antigens and DAMPs [32,99]. These chemicals indicate the existence of atypical cells to the immune system. Furthermore, PANoptosis stimulates inflammasomes, leading to the synthesis and secretion of inflammatory cytokines, including IL-1β and IL-18, via caspases [100]. These cytokines augment the immune response, facilitating anti-tumor activity. The liberated antigens and cytokines draw and stimulate immune cells, including dendritic cells, T cells, and NK cells. The heightened presence and activity of immune cells facilitates the immunogenic remodeling of the tumor microenvironment [101]. Recent studies indicate that the interplay of specific cytokines, including TNF and interferon-gamma (IFN-γ), might trigger PANoptosis in neoplastic cells, potentially resulting in decreased tumor size in preclinical models [102,103]. The findings indicate that PANoptosis may directly facilitate the eradication of colon cancer cells.

PANoptosis, like other regulated cell death mechanisms, can effectively generate highly immunogenic tumors. Research has shown that immunogenic PANoptotic cell death can alter an immunosuppressive environment and enhance innate immune responses. This is achieved by enhancing dendritic cell maturation and macrophage polarization via the generation of DAMPs [104]. Targeting PANoptotic cell death not only prevents immune evasion but also establishes a feedback loop for immunological activation, crucial for surmounting resistance in treatment-resistant cancers. Moreover, PANoptosis is positively correlated with the invasion of immune cells, including CD4+ T cells, CD8+ T cells, and NK cells, in the TME. This enhances the tumor-specific immune response. PANoptotic characteristics exhibit a positive connection with immune checkpoint markers such as CD4, CD274, CCL2, CXCR4, and LAG-3 [105,106]. PANoptosis significantly contributes to the immune response against tumors by promoting immune cell infiltration, enhancing tumor immunogenicity, and increasing the expression of immunological checkpoint regulators.

Further investigation into the relationships between these immunological variables and PANoptosis may yield innovative strategies to improve cancer therapy. The interplay between immunological elements and PANoptosis in cancer treatment presents significant therapeutic and prognostic potential.

## 5. Preclinical and Clinical Dimensions of PANoptosis in Colorectal Cancer

Interferon regulatory factor 1 (IRF1) is a transcription factor that conducts the function of interferons. It contributes to inflammation, innate and adaptive immunity, and tumor surveillance. IRF1 modulates the gene expression of guanylate-binding proteins, inducible nitric oxide synthase, and caspase-1 [107,108,109], which have roles in numerous inflammatory disorders. IRF1 has been demonstrated to facilitate the activation of the NLRP3 and AIM2 inflammasomes in response to microbial infection [109,110,111]. For instance, the detection of bacterial DNA by cGAS, followed by cGAS/STING-mediated type I IFN-dependent production of IRF1, stimulates the expression of guanylate-binding proteins. This results in the intracellular eradication of bacteria and the release of DNA [109]. Additionally, in fungal infections, the C-type lectin receptor pathway activates both MAPK and NF-κB signaling, resulting in the induction of IRF1 and the priming of inflammasomes. TLR signaling via adaptor molecules MyD88 and TRIF facilitates the effective activation of IRF1, which then stimulates IRGB10 expression, ultimately leading to antifungal action [110]. These inflammasomes have been linked to the emergence of preventive mechanisms against colon cancer [112,113,114]. The investigation of IRF1’s role in carcinogenesis through PANoptosis revealed that IRF1 substantially decreases CRC incidence in mice, and the induction of PANoptosis successfully inhibits AOM/DSS-induced colon cancer. Nonetheless, Irf1-deficient animals exhibited an atypical vulnerability to colitis-associated colon cancers [115]. The comparison of the production of pro-inflammatory cytokines and the death of colonic epithelial cells in Irf1-deficient mice compared to wild-type mice showed that there was no significant difference in the production of cytokines. However, the death of colon cells was significantly lower in Irf1-deficient mice. The decrease in colon cell mortality was linked to diminished activation of caspase-3 and -7, as well as a reduction in pyroptosis and necroptosis. All of these are supported by IRF1-regulated production of TNF-α and IFN-γ. This indicates that IRF1, as an upstream regulator of PANoptosis, promotes PANoptosis in colonic epithelial cells, hence safeguarding them from cancer [115]. Moreover, the loss of IRF1 in subepithelial myofibroblasts and fibroblasts may also facilitate enhanced colorectal carcinogenesis in whole-body knockout mice. The findings indicate that IRF1 could serve as a viable target in the modulation of many PCD pathways.

The PANoptosis sensor ZBP1 triggers cell death, while the RNA editor ADAR1 (adenosine deaminase acting on RNA 1) preserves a balance between cell death and survival [116]. The interplay between ADAR1 and ZBP1 is significant in the modulation of cellular death in neoplasms. The combination of IFN and NEI (nuclear export inhibitor) can stimulate ZBP1-mediated PANoptosis. ADAR1, however, obstructs this process by engaging with the Zα2 structural domain of ZBP1; hence, it prevents the interaction between ZBP1 and RIPK3 [116,117]. Adar1 knockout mice exhibit resistance to CRC growth, which can be counteracted by the deletion of the Zα2 domain of ZBP1 [116,118]. This evidence indicates that ADAR1 suppresses ZBP1-mediated PANoptosis and exerts a protumorigenic function.

Metabolic reprogramming is a defining hallmark of cancer cells [119]. Metabolic enzymes are crucial to metabolic reprogramming [120]. Their abnormal expression is intricately linked to carcinogenesis, tumor development, and chemotherapeutic sensitivity [120]. Iron–sulfur (Fe–S) clusters serve as essential cofactors for Fe–S proteins and participate in numerous physiological activities, including iron homeostasis, energy metabolism, and lipid production [121]. Fe–S clusters are markedly elevated during the fast proliferation of cancer cells [122]. In vivo CRISPR-Cas9 screening of metabolic enzyme genes revealed that the deletion of the rate-limiting enzyme in Fe–S cluster biogenesis, cysteine desulfurase (Nfs1), enhances the efficacy of antitumor therapy by elevating intracellular oxidative stress-induced PANoptosis, in conjunction with oxaliplatin [123]. NFS1 S293 phosphorylation-dependent inhibition of PANoptosis occurs following oxaliplatin therapy. The elevated expression of NFS1 in colorectal cancer patients correlates with unfavorable prognosis [124].

It has been discovered that the depletion of Wilms tumor 1-associating protein (WTAP; a m6A methyltransferase) in CRC cells caused PANoptosis in response to oxaliplatin treatment by increasing intracellular oxidative stress [125]. Additionally, the treatment with oxaliplatin increased the expression of WTAP, which, in turn, prevented PANoptosis by maintaining the expression of nuclear factor erythroid-2-related factor 2 (NRF2), a significant antioxidant response element, through a m6A-dependent mechanism. Furthermore, clinical data analysis of The Cancer Genome Atlas (TCGA) database and patient cohort studies have shown that high WTAP expression in CRC patients is associated with a poor prognosis and diminished benefit from standard chemotherapy [125].

A recent study utilized TCGA and GEO datasets to find differential lncRNAs linked to metastasis and PANoptosis in CRC [126]. Differentially expressed lncRNAs were utilized to establish a lncRNA–miRNA–mRNA network, and the functional and prognostic implications of these lncRNAs were further examined by various bioinformatics methods. The authors found the PANoptosis-related lncRNA SNHG7 linked to CRC metastases, chemoresistance, and prognosis. Consequently, lncRNA SNHG7 has been proposed as a possible predictive biomarker and therapeutic target for CRC [126].

Also using the TCGA database, the mRNA expression dataset was profiled in 404 CRC cases [127]. After identifying key genes associated with PANoptosis, a prognostic model (i.e., TIMP1, CDKN2A, CAMK2B, and TLR3) was developed that showed high predictive accuracy for CRC prognosis. A significant association was found between high PANoptosis risk scores and worse survival outcomes [127]. The finding highlights the potential of these genes as biomarkers for the diagnosis and prognosis of CRC.

Another study identified key PANoptosis-related genes (i.e., BCL10, CDKN2A, DAPK1, PYGM, and TIMP1) associated with CRC progression using multiple datasets [128]. They then defined the pathogenic regions of these genes and explored their relationship with the immune microenvironment and chemotherapeutic drug sensitivity, tumor progression genes, single-cell subpopulations, signaling pathways, transcription factor regulation, and miRNA regulatory networks in CRC. A total of 146 miRNAs have been identified through in silico analysis as modulating PANoptosis via post-transcriptional regulation of the BCL10, PYGM, CDKN2A, DAPK1, and TIMP1 genes; however, none have been validated in vitro or in vivo [128]. By using these complex results, they successfully constructed a new prognostic nomogram model for CRC. Several datasets have demonstrated the clinical relevance and prognostic value of key genes [128].

Table 2 presents a summary of the activities of RNA types that govern PANoptosis in colorectal carcinogenesis.

The analysis of differentially expressed genes linked to PANoptosis indicated a reduction in UNC5D gene expression in HCT116, HT29, and SW480 colon cancer cell lines [129]. An analysis of the TCGA database indicated that diminished expression of UNC5D correlates with inferior survival in CRC [129].

**Figure 4:**
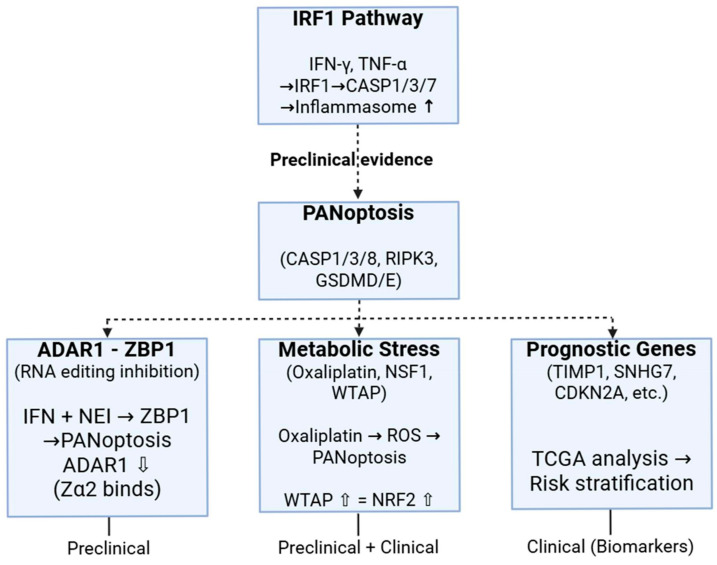
Schematic overview of PANoptosis pathways and their modulation in colorectal cancer. Upstream regulatory pathways—including IRF1 signaling, ADAR1-ZBP1 interactions, and metabolic stress—integrate to activate PANoptosis via the PANoptosome complex. Preclinical and clinical evidence highlights the therapeutic and prognostic relevance of targeting PANoptosis-related mechanisms in CRC. ADAR1: Adenosine deaminase acting on RNA-1; CASP: caspase; CDKN2A: cyclin-dependent kinase inhibitor 2A; GSDM: gasdermin; IFN: interferon; IRF1: Interferon regulatory factor 1; NSF1: cysteine desulfurase; NRF2: nuclear factor erythroid-2-related factor 2; RIPK: Receptor-interacting serine/threonine-protein kinase; ROS: reactive oxygen species; SNHG7: Small Nucleolar RNA Host Gene 7; TGCA: The Cancer Genome Atlas; TIMP1: metallopeptidase inhibitor 1; TNF: tumor necrosis factor; WTAP: Wilms tumor 1-associating protein; ZBP1: Z-DNA-binding protein 1; ↑ or ↓: enhanced or suppressed function. Figure was partly created with https://biorender.com/ (accessed on 12 May 2025).

As new discoveries about PANoptosis and its involvement in colorectal cancer continue to grow, it is becoming clear that PANoptosis could play an important role in predicting outcomes and treating the disease. Figure 4 illustrates the schematic representation of PANoptosis pathways and their regulation in colorectal cancer.

### Expression of Main Components of PANoptosis in Colorectal Cancer

**Figure 5:**
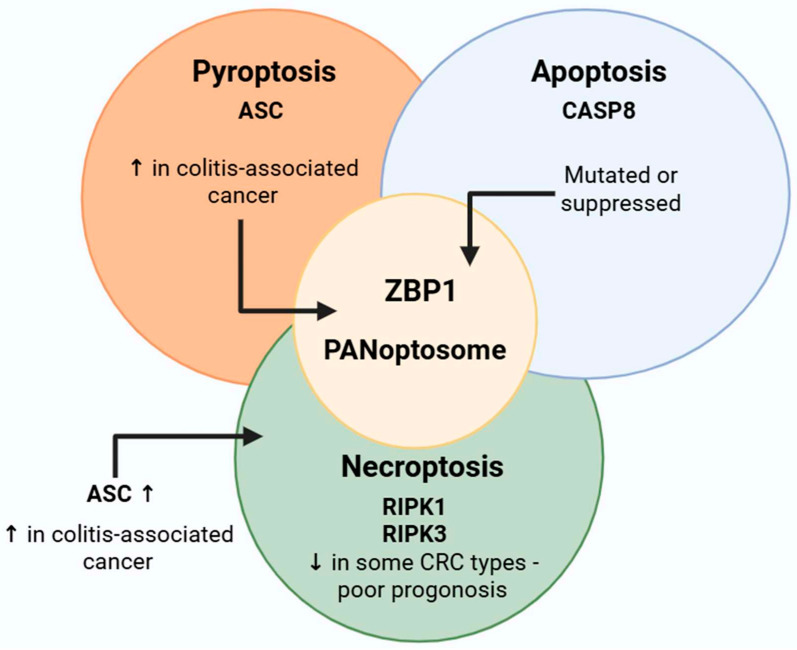
Key molecular players and crosstalk in PANoptosis in colorectal cancer. The diagram highlights the core components of apoptosis (CASP8), pyroptosis (ASC, CASP1), and necroptosis (RIPK1, RIPK3), with ZBP1 acting as a central upstream regulator. These molecules can exhibit either tumor-promoting or tumor-suppressive roles depending on the tumor microenvironment and inflammatory context. ASC: Apoptosis-associated speck-like protein containing a CARD; CASP: caspase; CRC: colorectal cancer; RIPK: Receptor-interacting serine/threonine-protein kinase; ZBP1: Z-DNA-binding protein 1; ↑ or ↓: enhanced or suppressed function. Figure was partly created with https://biorender.com/ (accessed on 12 May 2025).

The principal elements of PANoptosis—proteins implicated in the formation of the PANoptosome—may be expressed in various tumors, including colorectal cancer, and their function can be dual: either anti-tumor or supportive of tumor survival, contingent upon the setting. The schematic representation of key molecular players in PANoptosis and their overlap in colorectal cancer is visualized in Figure 5.

The ZBP1 gene is often expressed at higher levels in colon cancer cells and colon cancer, especially in situations involving inflammation like colitis-associated cancer [130,131,132]. The expression of ZBP1 may activate the PANoptosome by enlisting more adaptors, hence affecting the progression of colon cancer.

The expression of RIPK1 and RIPK3 is variable in CRC, absent in certain tumors and overactivated in others [133,134]. The lack of RIPK3 may correlate with a poorer prognosis [135,136].

CASP8 is often mutated or epigenetically suppressed in colorectal cancer [137,138,139]. In TRAIL-resistant DLD1 colon cancer cells, reduced stability and expedited degradation of caspase-8 have been seen [140]. Decreased levels of CASP8 may facilitate necroptosis or PANoptosis cell death [141,142].

In colon cancer, ASC levels or activity may be elevated in an inflammatory microenvironment [143,144,145]. The activation of ASC may trigger PANoptosis through inflammasome-associated caspases, such as CASP1.

The expression profile of PANoptosis components may hold prognostic and predictive value. It offers a chance to formulate focused therapy strategies, such as combination modulators of inflammasomes and necroptosis. Conversely, it may also be relevant in conjunction with immunotherapy, as PANoptosis represents an immunogenic variant of cell death and may hence augment the anti-tumor immune response.

## 6. Therapeutic Strategies and Targets Based on PANoptosis in Colorectal Cancer

The therapeutic implications of PANoptosis in CRC are now being explored through many avenues, primarily relying on preclinical models and expression data.

PANoptosis is an immunogenic form of cell death; it creates an inflammatory environment and promotes the activation of the anti-tumor immune response. A therapeutic target may be to sensitize tumor cells with agents that induce PANoptosis through activation of ZBP1, CASP8, RIPK3, or other key components [146,147,148]. This trait may be particularly important for tumors that are resistant to apoptosis (e.g., p53-mutant CRC) [149].

Pro-inflammatory cytokines (e.g., IL-1β and IL-18) and DAMP molecules generated during PANoptosis may enhance the efficacy of anti-PD-1/PD-L1 or anti-CTLA-4 immunotherapies [150]. Animal models have demonstrated enhanced T cell infiltration and tumor regression following the administration of PANoptosis inducers [151].

ZBP1 agonists are not yet available, but ZBP1 may be self-activated in inflammatory settings, e.g., in IBD-associated colorectal carcinoma [152,153]. In CRC patients, chemotherapy triggers the emergence of dsRNA and damaged DNA, which are detected by ZBP1 in normal colorectal tissues, indicating that ZBP1-mediated PANoptosis is activated by chemotherapy in these tissues. This process exacerbates the deleterious side effects of chemotherapy due to DNA damage. Consequently, ZBP1 could serve as a viable therapeutic target to alleviate chemotherapy-induced side effects [154].

Drugs can affect RIPK3 and CASP8. In certain colorectal cancers, CASP8 is inactivated, which could lead to the amplification of necroptosis and PANoptosis pathways [155]. This may represent a prospective therapeutic window for anticancer treatment.

A recent study examined the production of a BiFe_2_O_4_@Ag nanocomposite utilizing *Chlorella vulgaris* extract and its cytotoxic effects on colon cancer cells [156]. The nanocomposite treatment resulted in a substantial upregulation of the CASP8, BAX, and BCL2 genes. Moreover, caspase-3 activity was elevated, and apoptotic morphological alterations were noted [156].

The second mitochondria-derived activator of caspases (SMAC) protein suppresses IAP activity. Numerous small molecules that mimic SMAC function have been synthesized in the past two decades [157]. Inhibition of XIAP by SMAC mimetics promotes caspase-3 activation, whereas the inhibition of cIAPs enables the establishment of the RIPK1-dependent platform for caspase-8 activation that governs cell death. Consequently, SMAC mimetics constitute a promising therapeutic strategy to enhance the sensitivity of apoptosis-resistant malignancies to chemotherapy [157,158]. The genomic heterogeneity of colorectal malignancies raises the question of whether certain consensus molecular subtypes (CMS) may exhibit greater susceptibility to IAP inhibition than others. It has been demonstrated that IAP inhibition is a potential modulator of responses to oxaliplatin/5-FU in colorectal tumors of the CMS1 subtype and may also have promise in the CMS2 subtype [158].

RIPK3 may facilitate tumor proliferation in certain malignancies that are RIPK3-positive or overexpressing RIPK3 [26]. RIPK3 inhibitors can impede the emergence and advancement of malignant tumors. In the case of insufficient diet, the upregulation of RIPK3 is inhibited by the tumor suppressor gene TSC1 [159]. The reduction of gut microbiota through antibiotics or probiotics may diminish RIPK3 activation and expression. This process can lead to reduced intestinal epithelial cell necrosis and mitigated colonic inflammation [159].

OSW-1, extracted from the bulbs of *Ornithogalum saundersiae*, demonstrates significant anticancer efficacy [160]. The inhibitory impact of OSW-1 on colorectal cancer has been established by both in vitro and in vivo studies. In colon cancer cells, OSW-1 has been demonstrated to cause necroptosis through the RIPK1/RIPK3/MLKL signaling pathway, with this action mediated by the RIPK1-p62/SQSTM1 complex [161].

Necrostatin-1 is a particular inhibitor of necroptosis that functions by blocking the interaction between receptor-interacting protein (RIP)1 and RIP3 [162]. The efficacy of necrostatin-1 as an anti-inflammatory and antitumorigenic agent was examined in animal models of DSS-induced colitis and colitis-associated cancer [163]. Necrostatin-1 markedly diminished the clinical and histological severity of colitis. The injection of Necrostatin-1 reduced the elevation of RIP1 and RIP3 while augmenting the expression of caspase-8 in DSS-induced colitis. Furthermore, necrostatin-1 administration diminished the synthesis of pro-inflammatory cytokines and the release of extracellular HMGB1 in HT29 colon cancer cells undergoing active necroptosis. Moreover, the treatment of necrostatin-1 markedly inhibited tumor growth and progression via suppressing JNK/c-Jun signaling [163].

**Figure 6:**
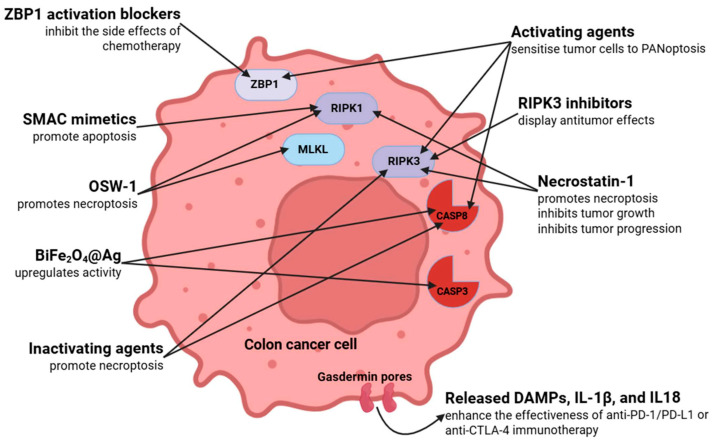
Potential PANoptosis-related therapeutic targets and mechanisms in colorectal cancer. ZBP1: Z-DNA-binding protein 1; SMAC: second mitochondria-derived activator of caspases; OSW-1: Orsaponin 1; DAMP: danger-associated molecular pattern; IL: interleukin; RIPK: receptor-interacting serine/threonine protein kinase; MLKL: mixed lineage kinase domain-like pseudokinase; CASP: caspase. Figure was partly created with https://biorender.com/ (accessed on 6 April 2025).

Potential anticancer therapeutic targets affecting molecules and processes involved in PANoptosis are summarized in Figure 6.

## 7. Future Perspectives

The capacity of PANoptosis to inhibit or mitigate cancer is increasingly recognized, and the proteins and regulatory elements governing PANoptosomes may be associated with tumor pathophysiology [164].

However, a number of technological challenges remain to be solved in investigating the role of PANoptosis in CRC. The task includes, in part, a precise understanding of the bypass mechanisms caused by complex and redundant signaling pathways, as well as mapping the different biological responses of cancer cells without and with TME [165].

Cancer cell heterogeneity leads to disparate responses to PANoptosis within the same tumor, attributable to variations in transcriptional activity, oxidative stress response, and antioxidant defenses [166]. Such variability hampers the formulation of universal medicines, as efficacy may vary among cancer types and even patients with identical cancers.

A significant future research avenue should focus on the correlation between PANoptosis and colorectal cancer stem cells (CSCs). Currently, we possess no significant data about the impact of PANoptosis on cancer cell resilience to stress and medicines. The influence of the tumor/inflammatory microenvironment, shaped by PANoptosis, on the survival and multiplication of cancer stem cells requires thorough investigation. Investigating the specific involvement of PANoptosis processes in the treatment resistance of CSCs is equally crucial.

Also, the complex mechanism of PANoptosis implies that the therapeutic risks and unwanted effects of manipulating PANoptosis need to be understood in detail.

The processes of PANoptosis and cell death encompass many intermolecular interactions. A comprehensive understanding of how these factors affect PANoptosis is essential for practical application.

## Figures and Tables

**Table 1:** Summary of key molecular components involved in PANoptosis pathways.

Pathway	Key Molecules	Role in PANoptosis	Reference
Apoptosis	CASP3, CASP7, CASP8, and CASP9	CASP3/7 execute apoptosis; CASP8 initiates apoptosis or inhibits necroptosis	[16,25]
Necroptosis	RIPK1, RIPK3, and MLKL	RIPK1/RIPK3 activate MLKL; MLKL disrupts membrane; inhibited by CASP8	[17,26,27]
Pyroptosis	CASP1, CASP4, CASP5, CASP11, ASC, and GSDMD	CASP1 activates IL-1β/IL-18 and cleaves GSDMD; ASC scaffolds inflammasome formation	[14,24]
PANoptosome scaffold	ZBP1, RIPK3, and ASC	ZBP1 senses stress/DNA and recruits RIPK3, ASC, and CASP8 to form the PANoptosome	[21,22,28,30]
Executioners shared across pathways	CASP1, CASP3, CASP8, GSDMD, GSDME, and MLKL	Mediate final membrane disruption and cell death	[23,24,29]

**Table 2:** The role of regulatory RNAs and mRNAs in the process of PANoptosis in CRC.

lncRNA/miRNA or mRNA	Function/Role	Reference
lncRNA SNHG7	Associated with CRC metastasis, chemoresistance, and prognosis; proposed as a predictive biomarker and therapeutic target.	[126]
miR-33ab; miR-34ac; miR-101; miR-187	Affect PANoptosis in CRC through post-transcriptional regulation of the BCL10 gene.	[128]
miR-15abc; miR-31; miR-133abc; miR-191	Influence PANoptosis in CRC via post-transcriptional modulation of the CDKN2A gene.	[128]
miR-23abc; miR-181abc; miR-217; miR-455-5p;	Modulate PANoptosis in CRC through post-transcriptional regulation of the DAPK1 gene.	[128]
miR-18ab; miR-19ab; miR-141; miR218	Affect PANoptosis in CRC via post-transcriptional modulation of the TIMP1 gene.	[128]
miR-1ab; miR-145; miR-193; miR210	Influence PANoptosis in CRC through post-transcriptional modulation of the PYGM gene.	[128]
TIMP1 (mRNA)	Part of a PANoptosis-related prognostic model; associated with poorer survival in CRC.	[127,128]
CDKN2A (mRNA)	Involved in CRC prognosis and progression; linked to immune microenvironment and drug sensitivity.	[127,128]
CAMK2B (mRNA)	Component of a PANoptosis-based prognostic model; contributes to CRC survival prediction.	[127]
TLR3 (mRNA)	Included in the prognostic model; high PANoptosis risk score correlates with worse CRC survival.	[127]
BCL10 (mRNA)	PANoptosis-related gene associated with CRC progression; involved in immune response and drug sensitivity.	[128]
DAPK1 (mRNA)	Plays a role in CRC and PANoptosis; potentially involved in signaling and therapeutic response.	[128]
PYGM (mRNA)	PANoptosis gene linked to CRC progression; shows prognostic value.	[128]

## Data Availability

No new data were created.

## Abbreviations

ADAR1: Adenosine deaminase acting on RNA-1; AIM2: Absent In Melanoma 2; Akt: protein kinase B; ALR: AIM2-like receptor; AOM: azoxymethane; ASC: Apoptosis-associated speck-like protein containing a CARD; Bcl-2: B-cell lymphoma 2; Bcl-xL: B-cell lymphoma-extra large; CASP: caspase; CCL2: Chemokine CC motif ligand 2; CDKN2A/B: cyclin-dependent kinase inhibitor 2A/B; cGAS: cyclic GMP-AMP synthase; cIAP: Cellular inhibitor of apoptosis; CLR: C-type lectin receptor; CMS: consensus molecular subtypes; CRC: colorectal cancer; CRISPR: clustered regularly interspaced short palindromic repeats; CTLA-4: Cytotoxic T-lymphocyte associated protein 4; CXCR4: C-X-C chemokine receptor type 4; CSC: cancer stem cell; DAMP: danger-associated molecular pattern; DAPK: Death-associated protein kinase 1; DNA: Deoxyribonucleic acid; dsRNA: double-stranded RNA; DSS: dextran sulfate sodium; ERK: extracellular signal-regulated kinase; GEO: Gene Expression Omnibus; GSDND/E: gasdermin D/E; HMGB1: high-mobility group protein 1; IFN: interferon; IL: interleukin; IRF1: Interferon regulatory factor 1; JNK: c-Jun N-terminal kinase; LAG-3: Lymphocyte-activation gene 3; lncRNA: long non-coding RNA; MAPK: Mitogen-activated protein kinase; MDSC: myeloid-derived suppressor cell; miRNA: microRNA; MLKL: Mixed lineage kinase domain like pseudokinase; mRNA: messenger RNA; MSI-H: Microsatellite instability-high; NEI: nuclear export inhibitor; NF-kB: nuclear factor-kB; Nfs1: cysteine desulfurase; NLR: NOD-like receptor; NLRP3: NLR family pyrin domain containing 3; NRF2: nuclear factor erythroid-2-related factor 2; OspC1/3: outer Shigella protein C1/3; OspD3: outer Shigella protein D3; OSW-1: Orsaponin 1; p53: Tumor protein P53; PAMP: pathogen-associated molecular pattern; PARP: Poly (ADP-ribose) polymerase; PCD: programmed cell death; PD-1: Programmed cell death protein 1; PD-L1: Programmed Death-Ligand 1; PI3K: Phosphoinositide 3-kinase; PRR: pattern recognition receptor; PYGM: glycogen phosphorylase, muscle associated; RIP1/3: Receptor interacting protein 1/3; RIPK1/3: Receptor-interacting serine/threonine-protein kinase 1/3; RLR: Rig-like receptor; RNA: Ribonucleic acid; ROS: reactive oxygen species; SMAC: second mitochondria-derived activator of caspases; SNHG7: Small Nucleolar RNA Host Gene 7; SQSTM1: Sequestosome 1; STAT3: Signal transducer and activator of transcription 3; STING: Stimulator of interferon genes; TAM: tumor-associated macrophage; TGCA: The Cancer Genome Atlas; TGF: transforming growth factor; Tim-3: T-cell immunoglobulin and mucin-domain containing-3; TIMP1: metallopeptidase inhibitor 1; TLR: Toll-like receptor; TME: tumor microenvironment; TNF: tumor necrosis factor; TRAIL: TNF-related apoptosis-inducing ligand; TSC1: tuberous sclerosis complex 1; UNC5D: Unc-5 Netrin Receptor D; VEGF: vascular endothelial growth factor; VirA: virulence factor A; WTAP: Wilms tumor 1-associating protein; XIAP: X-Linked Inhibitory Apoptosis Protein; ZBP1: Z-DNA-binding protein 1.
